# Osteoblast-specific down-regulation of NLRP3 inflammasome by aptamer-functionalized liposome nanoparticles improves bone quality in postmenopausal osteoporosis rats

**DOI:** 10.7150/thno.95423

**Published:** 2024-06-24

**Authors:** Lijun Xu, Jie Zhu, Lingjun Rong, Huinan Yang, Bin Wang, Shuai Lu, Lingxiao Zhang, Fuyi Li, Shihua Yang, Zhifang Wang, Chong Li, Xiao Hu, Ruoyun Liu, Lili Zheng, Hongjian Liu, Haohao Zhang, Yanling Liu, Di Zhao, Shuiying Zhao, Lun Zhang, Yingbo Jia, Shiyu Liang, Zhikang Guo, Xixiu Xie, Ruitian Liu, Lixia Zhang

**Affiliations:** 1 Department of Endocrinology, the First Affiliated Hospital of Zhengzhou University, Zhengzhou 450052, China.; 2 State Key Laboratory of Biochemical Engineering, Institute of Process Engineering, Chinese Academy of Sciences, Beijing 100190, China.; 3 Key Laboratory of Biopharmaceutical Preparation and Delivery, Chinese Academy of Sciences, Beijing 100190, China.; 4 University of Chinese Academy of Sciences, Beijing 100049, China.; 5 Department of Geriatric Endocrinology, the First Affiliated Hospital of Zheng Zhou University, Zhengzhou 450052, China.; 6 Key Laboratory of Novel Targets and Drug Study for Neural Repair of Zhejiang Province, School of Medicine, Zhejiang University City College, Hangzhou 310015, China.; 7 College of Life Science, Capital Normal University, Haidian District, Beijing 100048, China.; 8 School of Biomedicine, Beijing City University, Beijing 100094, China.; 9 Department of Orthopedics, the First Affiliated Hospital of Zhengzhou University, Zhengzhou 450052, China.

**Keywords:** postmenopausal osteoporosis, NLRP3 inflammasome, diagnosis, osteoblast-specific aptamer, lipid nanoparticles

## Abstract

**Rationale:** NLRP3 inflammasome is critical in the development and progression of many metabolic diseases driven by chronic inflammation, but its effect on the pathology of postmenopausal osteoporosis (PMOP) remains poorly understood.

**Methods:** We here firstly examined the levels of NLRP3 inflammasome in PMOP patients by ELISA. Then we investigated the possible mechanisms underlying the effect of NLRP3 inflammasome on PMOP by RNA sequencing of osteoblasts treated with NLRP3 siRNA and qPCR. Lastly, we accessed the effect of decreased NLRP3 levels on ovariectomized (OVX) rats. To specifically deliver NLRP3 siRNA to osteoblasts, we constructed NLRP3 siRNA wrapping osteoblast-specific aptamer (CH6)-functionalized lipid nanoparticles (termed as CH6-LNPs-siNLRP3).

**Results:** We found that the levels of NLRP3 inflammasome were significantly increased in patients with PMOP, and were negatively correlated with estradiol levels. NLRP3 knock-down influenced signal pathways including immune system process, interferon signal pathway. Notably, of the top ten up-regulated genes in NLRP3-reduced osteoblasts, nine genes (except Mx2) were enriched in immune system process, and five genes were related to interferon signal pathway. The *in vitro* results showed that CH6-LNPs-siNLRP3 was relatively uniform with a dimeter of 96.64 ± 16.83 nm and zeta potential of 38.37 ± 1.86 mV. CH6-LNPs-siNLRP3 did not show obvious cytotoxicity and selectively delivered siRNA to bone tissue. Moreover, CH6-LNPs-siNLRP3 stimulated osteoblast differentiation by activating ALP and enhancing osteoblast matrix mineralization. When administrated to OVX rats, CH6-LNPs-siNLRP3 promoted bone formation and bone mass, improved bone microarchitecture and mechanical properties by decreasing the levels of NLRP3, IL-1β and IL-18 and increasing the levels of OCN and Runx2.

**Conclusion:** NLRP3 inflammasome may be a new biomarker for PMOP diagnosis and plays a key role in the pathology of PMOP. CH6-LNPs-siNLRP3 has potential application for the treatment of PMOP.

## Introduction

Postmenopausal osteoporosis (PMOP), the most common type of osteoporosis, is a global health issue with a heavy financial burden 1. PMOP is characterized by decreased bone density and the deterioration of bone microarchitecture, leading to increased bone fragility and a higher risk of fracture 2-4. This condition affects the health-related quality of life for many women. Moreover, osteoporotic fractures are usually linked with considerable mortality 5. Since fractures can occur without prior symptoms, many individuals fail to receive a timely diagnosis and effective therapy in the early phase of osteoporosis. Therefore, exploration the pathogenesis of PMOP will facilitate the early diagnosis and treatment of the disease.

PMOP is caused by an imbalance between bone formation by osteoblast and bone resorption by osteoclast, which is often the result of estrogen deficiency 6. Previous reports showed that estrogen deficiency led to increased serum levels of tumor necrosis factor alpha (TNF-α) and interleukin-6 (IL-6) in ovariectomized (OVX) animals compared to the control group 7. NLRP3 inflammasome can active nuclear factor kappa B (NF-κB) signaling, upregulate the production of IL-1β, TNF-a, and matrix metalloproteinase-3 (MMP-3), and thus degrade cartilage in osteoarthritis 8. Snouwaert et.al reported that an NLRP3 mutation induced inflammation and caused osteoporosis and arthropathy in humanized mice 9. Moreover, NLRP3 may contribute to bone loss caused by bacterial infection 10. The above results indicated that NLRP3 may play an important role in PMOP, but the exact mechanisms underlying the effect of NLRP3 inflammasome on the pathology of PMOP are poorly understood. NLRP3 inflammasome is a vital component of the natural immune system. The NLRP3 inflammasome contains NLRP3, apoptosis-associated speck-like protein containing a CARD (ASC) and pro-caspase-1. Activated NLRP3 can cleave pro-caspase-1 into p20 and p10 subunits, inducing the maturation and release of pro-inflammatory cytokines such as pro-IL-1β and IL-18 11. NLRP3 inflammasome may regulated immune system to trigger the pathology of PMOP.

Osteoporosis drugs are often given at high doses or frequencies to counter their fast clearance by the body's excretory system and limit penetration into bone tissue, which may cause side effects. Recently, nanoparticles as drug delivery system applied to bone diseases has attracted increasing attention 12. The nanoparticles may deliver drugs to target tissues, optimize the drug doses, prevent biodegradation, decrease exposure to non-target cells, and avoid system toxicity 13, 14. Nanoparticles also extend medication intervals, leading to better patient compliance. There are many types of nanocarriers, including organic, inorganic and hybrids; among these, lipid nanoparticles (LNPs) are the most common for targeted drug delivery and have been successfully translated into clinical applications 15. In this study, we firstly examined the levels of NLRP3 in patients with PMOP, investigated the possible mechanisms underlying the effect of NLRP3 inflammasome on PMOP by reducing NLRP3 expression, and then we developed NLRP3 siRNA-containing and osteoblast-specific aptamer (CH6, 5'-AGTCTGTTGGACCGAATCCCGTGGACGCACCCTTTGGACG-3') modified LNPs (termed as CH6-LNPs-siNLRP3), to increase the specific deliverance to osteoblasts, and investigated the effect of LNPs on OVX rats.

## Methods

### Subjects and sample collection

This study was approved by the Institutional Review Board of the First Affiliated Hospital of Zhengzhou University Ethics Committee (2021-KY-1262-002). All subjects signed written informed consent before their enrolment in the study. Postmenopausal women had a T score of ≤ - 2.5 and ≥ - 4 at the lumbar spine (L1-L4), total hip, or femoral neck or were at high risk for fracture based on the Fracture Risk Assessment Tool (FRAX) criteria 16 and diagnosed as PMOP were included in the study. Key exclusion criteria were as follows: metabolic bone disease, endocrine and metabolic diseases such as germinal aplasia, hyperthyroidism, hyperparathyroidism, Cushing syndrome, diabetes mellitus, pituitary diseases, severe chronic kidney disease, malignant tumor, severe gastrointestinal diseases, hematological system diseases, connective tissue diseases, long-term confined to bed, use of drugs affecting bone metabolism. The healthy subjects included in the study had a T score of≥-1.0 at the lumbar spine (L1-L4), total hip, and femoral neck and at low risk for fracture based on the Fracture Risk Assessment Tool (FRAX) criteria. We collected serum samples from patients with PMOP (n = 55) with a median age of 62.6 years (range, 46-84 years) and 29 healthy control subjects with a median age of 63.4 years (range, 44-81 years). Patient information, including age, sex and clinical diagnosis, was shown in Table S1. The levels of estradiol (E2), NLRP3 in human sera were detected by E2 (Beyotime, China), NLRP3 (Solarbio, China) ELISA Kit according to the manufacturer's instructions, respectively.

### Animals

All animal experiments were performed in accordance with the China Public Health Service Guide for the Care and Use of Laboratory Animals. Experiments involving rats and protocols were approved by the Institutional Animal Care and Use Committee of Tsinghua University (15-LRT1). 12-week-old female SD rats were purchased from the Beijing Vital River Laboratory Animal Technology Co., Ltd and maintained with access to food and water ad libitum in a colony room kept at 22 ± 2 °C and 50 ± 5% humidity, under a 12:12 light/dark cycle.

### Osteoblasts isolated from the rat calvaria

The osteoblasts were isolated from the calvaria of rats with and without OVX (WT) by enzyme digestion as previously described with minor modifications 17, 18. After the rats were sacrificed, the calvaria were obtained under aseptic conditions, further digested with 0.25% trypsin for 20 min at 37 °C, and followed by digesting with 0.2% collagenase type II for 30 min, then calvaria were cut into small pieces, and digested with 0.2% collagenase type II for 60 min. The digested cell suspension was centrifuged at 300 g/min for 5 min, and the cell pellet was the osteoblasts. These osteoblasts were cultured in DMEM/F12 medium (Gibco, USA) containing 10% fetal bovine serum (Gibco, USA), 100 U/ml penicillin and 100 µg/ml streptomycin (Sigma, USA) and used in knockdown efficiency of NLRP3 siRNA, MTT experiments, Alizarin Red S and Alkaline phosphatase (ALP) staining.

### NLRP3 siRNA transfection

Osteoblast cells isolated from WT rats were plated at 70% confluence and transfected with NLRP3 siRNA-1, siRNA-2 and siRNA-3 using Lipofectamine 3000 (Thermo Fisher Scientific, USA) according to the manufacturer's protocol. The NLRP3 siRNA in this study are described as follows:

NLRP3-siRNA-1:

5'-TGCATGAACTCTTGACCATTTTCAAGAGAAATGGTCAAGTCATGCTTTTTTC-3' and 5'-TCCAGGATCCTCTTCATTTCAAGAGAATGAGGAAGAGGATCCTGGTTTTTTC-3'.

NLRP3-siRNA-2:

5'-TCGAGAAAAAAGCATGAACTCTTGACCATTTCTCTTGAAAATGGTCAAGAGTTCATGCA-3' and 5'-TCGAGAAAAAACCAGGATCCTCTTCCTCATTCTCTTGAAATGAGGAAGAGGATCCTGGA-3'.

NLRP3-siRNA-3:

5'-TGCCTCATCCGAAAGAAGTTTTCAAGAGAAACTTCTTTCGGATGAGGCTTTTTTC-3' and 5'-TCGAGAAAAAAGGGTCCTCATCCGAAAGAAGTTTCTCTTGAAAACTTCTTTCGGATGAGGCA-3'.

### Real-time quantitative PCR (qPCR) analysis

RNA was extracted from cell lysates using the RNeasy Lipid Tissue kit (Qiagen, #74804) according to the manufacturer's instructions. cDNA was prepared from total RNA using the PrimeScript RT-PCR kit (Takara, #RR037Q). Relative gene expression of the cDNA was assayed using a 7500 Fast real-time PCR instrument (Applied Biosystems) with SYBR Select Master Mix (Applied Biosystems, #4472908), qPCR data were analyzed by the ΔΔCT method by normalizing the expression of each gene to housekeeping gene GAPDH and then to the control groups. The primers used in this study are described in Table 1.

### Isolation and culture of mesenchymal stem cells and Osteogenic differentiation

The bones of female OVX SD rats were collected under sterile conditions, and then all the bones were cut at both ends. The bone marrow from each bone was collected by flushing the bone with Dulbecco's Modified of Eagle's Medium (DMEM) (Gibco, USA) containing 1000 u/ml Penicillin G. After filtering, the cells were centrifuged at 1500 ×g for 5 min. The cell pellet was then resuspended in DMEM/F12 medium containing 10% fetal bovine serum, 100 U/ml penicillin and 100 µg/ml streptomycin. Then the cells were plated in T25 tissue culture flasks at a density of 1 × 10^6^ cells and maintained in a tissue culture incubator at 37 °C and 5% CO_2_. The medium was replaced every third day afterwards. Cells were subcultured using 5 ml trypsin/EDTA (Sigma, USA) when the flasks reached 90% confluence. Cells were then cultured in osteogenic medium (DMEM/F12 (Gibco, USA), containing 10% fetal bovine serum (Gibco, USA), 0.05 mM ascorbate, 1 µM dexamethasone and 10 mM β-glycerophosphate) for 4 weeks.

### High-throughput sequencing of RNAs (RNA-seq) and data analysis

Osteoblasts differentiated from mesenchymal stem cells were cultured in DMEM/F12 medium (Gibco, USA), containing 10% fetal bovine serum (Gibco, USA). Then the cells were plated at 70% confluence and treated with NLRP3 siRNA-3 and negative control siRNA using lipofectamine 3000 for 48 h. Total RNA was isolated from osteoblasts using a RNeasy mini kit (Qiagen, German). For each sample, 1 μg total RNA was used for library preparation. In brief, the poly(A) mRNA isolation was performed using Oligo(dT) beads and the mRNA fragmentation was performed using divalent cations and high temperature. Priming was performed using Random Primers. First-strand cDNA and the second-strand cDNA were synthesized. The purified double-stranded cDNA was then treated to repair both ends and add a dA-tailing in one reaction, followed by a T-A ligation to add adaptors to both ends. Size selection of adaptor-ligated DNA was then performed using DNA Clean Beads. Each sample was then amplified by PCR using P5 and P7 primers and the PCR products were validated. Then libraries with different indexes were multiplexed and loaded on an Illumina Novaseq instrument for sequencing using a 2x150 paired-end configuration according to manufacturer's instructions. DESeq2 was used to identify differentially expressed genes (DEGs) and the significant genes with fold change>1.5, and adjusted p value<0.05 were used for subsequent analysis and hierarchical clustering was performed as described 19.

### Preparation and characterization of lipids and lipoplexes

#### Synthesis of DSPE-PEG-CH_6_

CH6 which was synthesized by Shengong Biological Engineering Co.,LTD was conjugated with DSPE-PEG2000-MAL (Avanti Polar Lipids, USA) at room temperature for 72 h under gentle stirring, the resulting reaction solution was dialyzed against distilled water in a dialysis membrane with a molecular weight cut-off of 2000 Da for 48 h, then freeze-dried and stored at -20 °C. The conjugations were confirmed by agarose gel electrophoresis.

### Preparation of CH6-LNPs-siNLRP3

CH6-LNPs were prepared using a modified lipid film hydration technique. Briefly, DOTAP (Avanti Polar Lipids, USA), DSPC (Aladdin, China), DSPE-PEG-MAL-CH6, and cholesterol (Solarbio, China) with a molar ratio of 1:2:1:0.5 were dissolved in 1 ml chloroform (total concentration of 10 mg/ml). The solvent was then eliminated by rotary evaporation at 40 °C for 6 h to form a lipid film. The lipid film was then resuspended with PBS (pH 7.4) to obtain a 1 mg/mL lipid suspension, and followed by sonication in an ultrasonic bath sonicator. The formed CH6-LNPs were extruded through 400 nm and 100 nm polycarbonate membranes, respectively, using a Mini-Extruder (Avanti Polar Lipids, USA). The prepared CH6-LNPs were lyophilized for 24 h to achieve a preservable lipid powder for future use. To determine whether CH6 was grafted on LNPs, CH6-LNPs were applied to 3% agarose gel (Solarbio, China), and stained by ethidium bromide (Thermo Fisher Scientific, USA).

The optimal N:P ratio (a molar ratio) of LNPs-siNLRP3 was determined by gel retardation assay using 3% agarose gel (Solarbio, China) containing 0.5 μg/mL ethidium bromide. The hydrodynamic sizes and zeta potentials of the CH6-LNPs-siNLRP3 were characterized at room temperature and at a scattering angle of 90° by dynamic light scattering (DLS) using Zetasizer Nano ZSP (Malvern).

### Transmission Electron Microscopy (TEM) Assay

CH6-LNPs-siNLRP3 were applied to 200 mesh copper grids for 5 min, blotted with filter paper, and negatively stained with 2% uranyl acetate for 1 min, then blotted and air dried. CH6-LNPs-siNLRP3 were imaged in a Hitachi TEM system at 100 kV at 60,000 × magnification.

### The NLRP3 knockdown efficiency of CH6-LNPs-siNLRP3

Osteoblasts isolated from OVX rats were diluted in DMEM/F12 medium containing 10% fetal bovine serum, 100 U/ml penicillin and 100 µg/ml streptomycin and plated in 6-well plates at a density of 1x10^6^ cells/ml. One to two days later, the cells reached 70% confluence and were incubated with 40 nM NLRP3 siRNA encapsulated in CH6-LNPs (160 nM CH6) or 40 nM NLRP3 siRNA in lipofectamine 3000. Osteoblasts isolated from OVX rats or WT rats transfected with negative control siRNA were used as controls. 72 h later, relative gene expression of NLRP3, IL-1β, IL-18, Gbp2, STAT1, NF-κB, ALP, Sp7, Smad1, Smad5, Runx2 were analyzed by qPCR analysis.

### Alizarin Red S and ALP staining

Osteoblasts isolated from OVX rats were plated in 6-well plates at a density of 1x10^6^ cells/ml. One to two days later, the cells reached 70% confluence and were incubated with 40 nM NLRP3 siRNA encapsulated in CH6-LNPs (160 nM CH6) or 40 nM NLRP3 siRNA in lipofectamine 3000. Osteoblasts isolated from OVX rats or WT rats transfected with negative control siRNA were used as controls. 72 h later, cells were washed with PBS and fixed in 4% paraformaldehyde for 10-15 min for Alizarin Red S and ALP staining. For Alizarin Red S staining, 1% ARS (pH 4.2) (Beijing Solarbio Science & Technology Co.,Ltd.) was added to each flask. The plates were incubated at room temperature for 20 min, and washed four times with ddH_2_O, shaking for 5 min each time, then observed under a microscope. ALP staining was performed with a staining kit according to the manufacturer's instructions (Beijing Solarbio Science & Technology Co.,Ltd).

### MTT

MTT assay was used to detect the cytotoxicity of CH6-LNPs-siNLRP3. Briefly, 293T and osteoblast cells were maintained in DMEM or DMEM/F12 medium containing 10% fetal bovine serum, 100 U/ml penicillin, and 100 µg/ml streptomycin, respectively, in a 5% CO_2_ atmosphere at 37 °C. Cells were harvested from flasks and plated in 96-well polystyrene plates with approximately 8000 cells/ 100 μL of medium per well. After 12 h, cells were treated with a series of concentration gradients of samples for 72 h. Cell viability was determined by adding 25 μL of 5 mg/mL MTT (Sigma) to each well. After 3 h incubation at 37 °C, 150 μL of DMSO was added. The absorbance at 570 nm and 630 nm was measured by a SpectraMax M5 microplate reader (Molecular Devices, LLC, Sunnyvale, CA, USA). Averages from six replicate wells were used for each sample and control, and each experiment was repeated thrice. Cell viability was calculated by dividing the absorbance of wells containing samples (corrected for background) by the absorbance of wells containing medium alone (corrected for background).

### *In vitro* siNLRP3 release

Release behavior experiment of siNLRP3 was carried out according to previous reports with minor modifications 20, 21. 50 μl CH6-LNPs loaded with Cy3-labeled siNLRP3 were diluted with nuclease-free pH 6.5 PBS to a total volume of 500 μl and placed in 0.5-ml Amicon filter tubes. The tubes were maintained at 37 °C with gentle shaking for 1, 2, 4, 8, 16, 32, and 64 h. After incubation, the tubes were centrifuged at 5000 g for 30 min at 4 °C, and the flow-through fractions were collected. The volume of the flow-through was 0.45 ml. At each time point, except for the 64-h mark, 0.45 ml of fresh PBS buffer was added back into the tube. The amount of siNLRP3 in the collected flow-through fractions was measured using a fluorescence assay. The percentage release was calculated by the subtraction method, and the cumulative release rate was determined to establish the percentage release at various time points.

### Hemolysis assay

1 ml of whole rat blood sample was extracted from tail vein and stabilized with Heparin. Then the blood was added to 2 ml Dulbecco's phosphate-buffered saline (D-PBS) and centrifugated at 10, 000 g for 5 min to pellet red blood cells (RBCs). After washed five times with 5 ml of D-PBS, the RBCs were finally diluted to 10 ml with D-PBS. 0.2 ml of diluted RBC suspension was further incubated with 0.8 ml of the nanoparticle D-PBS suspension at a final nanoparticle concentration of 2, 10, 50, 100, 200 or 400 μg/ml (test group), D-PBS (negative group), and distilled water (positive group). Every group was represented for four tubes. After incubation at room temperature for 4 h and centrifugation for 5 min at 10, 016g, 100 μl of the supernatant of all samples was transferred to a 96-well plate and the absorbance was measured by a SpectraMax M5 microplate reader at 577 nm with a reference wavelength of 655 nm. The hemolytic degree was expressed by the hemolytic ratio as the following formula: hemolysis ratio = (OD(test) - OD(negative control))/(OD(positive control) - OD(negative control)) × 100%.

### Tissue distribution of siRNA

Tissue distribution of siRNA was performed according to previously established protocols 22. After the rats were sacrificed, heart, liver, spleen, lung, kidney and femur were cleaned with 0.9% normal saline, dried with filter paper. Fluorescence imaging of the Cy3-labeled siNLRP3 distribution in tissues was performed using a PerkinElmer ®IVIS Spectrum imaging system and fluorescence intensity in different organs was measured by Living Image®4.4.

### Drug administration

12-week-old female SD rats were purchased and allowed to acclimatize for 1 week. A fake operation was performed in the WT group as a control, and the other five groups (n=3) underwent bilateral OVX under anesthesia with pentobarbital sodium. After 8 weeks, OVX SD rats were treated with PBS, siNLRP3, LNPs-siNLRP3, CH6-LNPs-siCtrl and CH6-LNPs-siNLRP3 via tail vein injections at a single dose of 1.0 mg/kg siNLRP3 encapsulated in LNPs-based carriers, respectively, once a week for 6 weeks. WT were treated with PBS. The left femurs of rats were collected.

### Micro-computerized Tomography Analysis

Left femur of OVX rats were collected as previously described, and then they were examined by micro-computerized tomography (microCT) scanning (Quantum FX) 22. A refined volume of interest was generated 0.5 mm above the growth plate of the distal femur and 1 mm in height according to previous reports 23, 24. Bone parameters, including bone mineral density (BMD), relative bone volume (BV/TV), trabecular number (Tb.N), trabecular thickness (Tb.Th), and trabecular spacing (Tb. Sp) were analyzed.

### Bone morphology detected by hematoxylin and eosin (HE) and Masson staining

After removal of surface soft tissue, femurs were decalcified in 10% (w/v) EDTA (disodium salt) for about 3 months, embedded in paraffin, and then cut into 5-μm-thick sections. Subsequently, sections were stained with HE, and Masson to evaluate bone formation. Images were taken using Aperio Scanscope (Mt Waverley, VIC, Australia), and bone histomorphometry analyses were quantified using BIOQUANT OSTEO software (Bioquant Image Analysis Corporation, Nashville, TN, USA).

### Immunofluorescence (IF)

5 μm sagittal paraffin-embedded sections were washed in 20 mM PBS and then treated briefly with 80 % (vol/vol) methanol containing 0.3% H_2_O_2_ to prevent endogenous peroxidation. The sections were then blocked with 10% normal goat serum or donkey serum to prevent nonspecific protein binding. Subsequently, the sections were incubated with the primary antibodies overnight at 4 °C, and followed by corresponding fluorescently conjugated secondary antibodies, respectively, and imaged on a Leica TCS SP8 confocal microscope. All images were analyzed by Image J software.

The following primary antibodies were used for IF assay in this study: anti-Runx2 antibody (#AF2593, Beyotime, 1:100), anti-OCN antibody (#AF6297, Beyotime, 1:200), anti-IL-1β antibody (#AF5103, Affinity, 1:50), anti-IL-18 antibody (#AF5207, Beyotime, 1:50), anti-NLRP3 antibody (#AF2155, Beyotime, 1:50).

### HE staining of livers and kidneys

HE staining of livers and kidneys from CH6-LNPs-siNLRP3-treated rats were performed as follows. Livers and kidneys of rats were fixed in 4% paraformaldehyde at 4 °C for 24 h and processed for paraffin-embedded sections. 5 μm sagittal paraffin-embedded sections were dewaxed, and incubated with hematoxylin for 2 min, followed by washing with distilled water. Then sections were differentiated in differentiation solution for 10 s, and rinsed with water (two times for 3-5 min each). The sections were counterstained with eosin for 1 min, and quickly decolored in an alcohol series (70%, 95%, and 100%; 3 s each), followed by soaking in 100% alcohol for 1 min and finally immersed in xylene (two times for 1 min each). Images were acquired by an Olympus IX73 inverted microscope with DP80 camera.

### Statistical analysis

Data were analyzed with GraphPad Prism v.8.0.1. Each figure legend denotes the statistical test used. All data are represented as mean ± SD unless indicated otherwise, statistical significance was assessed using student's t-test or one-way ANOVA followed by Dunnett's test. *P* < 0.05 was considered statistically significant.

## Results

### The levels of NLRP3 inflammasome are significantly increased in patients with PMOP

**Figure 1:**
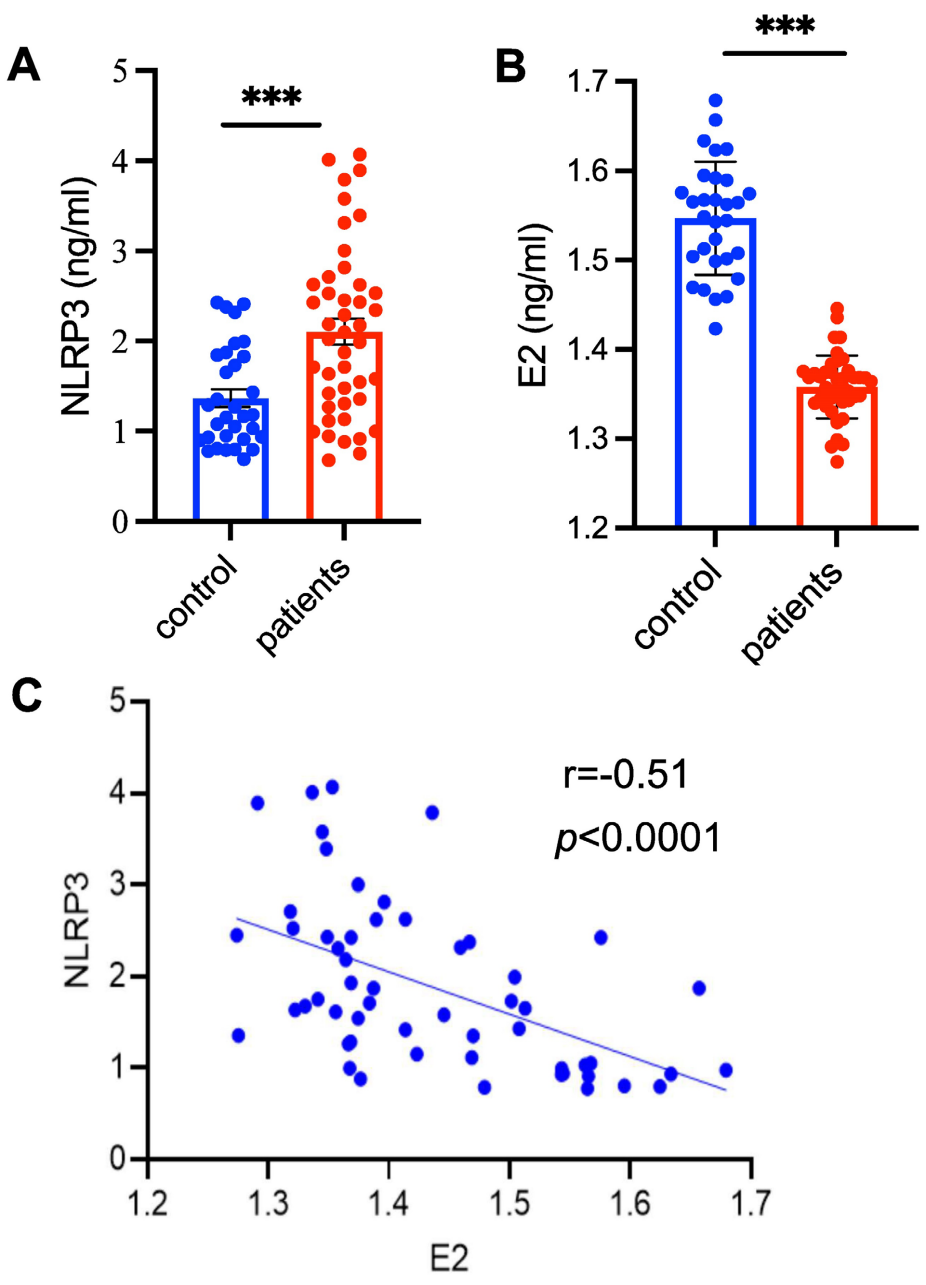
** The levels of NLRP3 inflammasome are significantly increased in patients with PMOP. (A-B)** The levels of NLRP3 inflammasome **(A)** and estradiol (E2) **(B)** in the patients with PMOP (n = 55) and healthy controls (n = 29). Data are means ± SD. **(C)** The levels of NLRP3 inflammasome in patients with PMOP were negatively correlated with E2 levels. Data includes both the healthy controls (n = 21) and PMOP patients (n = 34). For A and B, Statistical significance was calculated by Student t-test (Compared with Control, ***P < 0.001). For C, Statistical significance was analyzed by Pearson Correlation.

To detect the levels of NLRP3 inflammasome in patients with PMOP, the serum samples collected from patients with PMOP (n = 55) and 29 healthy control subjects were used for ELISA assay (Table S1). The levels NLRP3 inflammasome were significantly higher in patients with PMOP compared with of the control population (Figure 1A). Consistent with previous report 25, the levels of E2 were obviously lower in patients with PMOP (Figure 1B). We also found that the levels of NLRP3 inflammasome were negatively correlated with the E2 variable (*r* = -0.51, *P* **<** 0.0001) (Figure 1C). These results indicate that NLRP3 inflammasome may contribute to the pathology of PMOP and may be a new biomarker for PMOP diagnosis.

### Reduction of NLRP3 expression in osteoblasts regulates immune system process and interferon signal pathway

**Figure 2:**
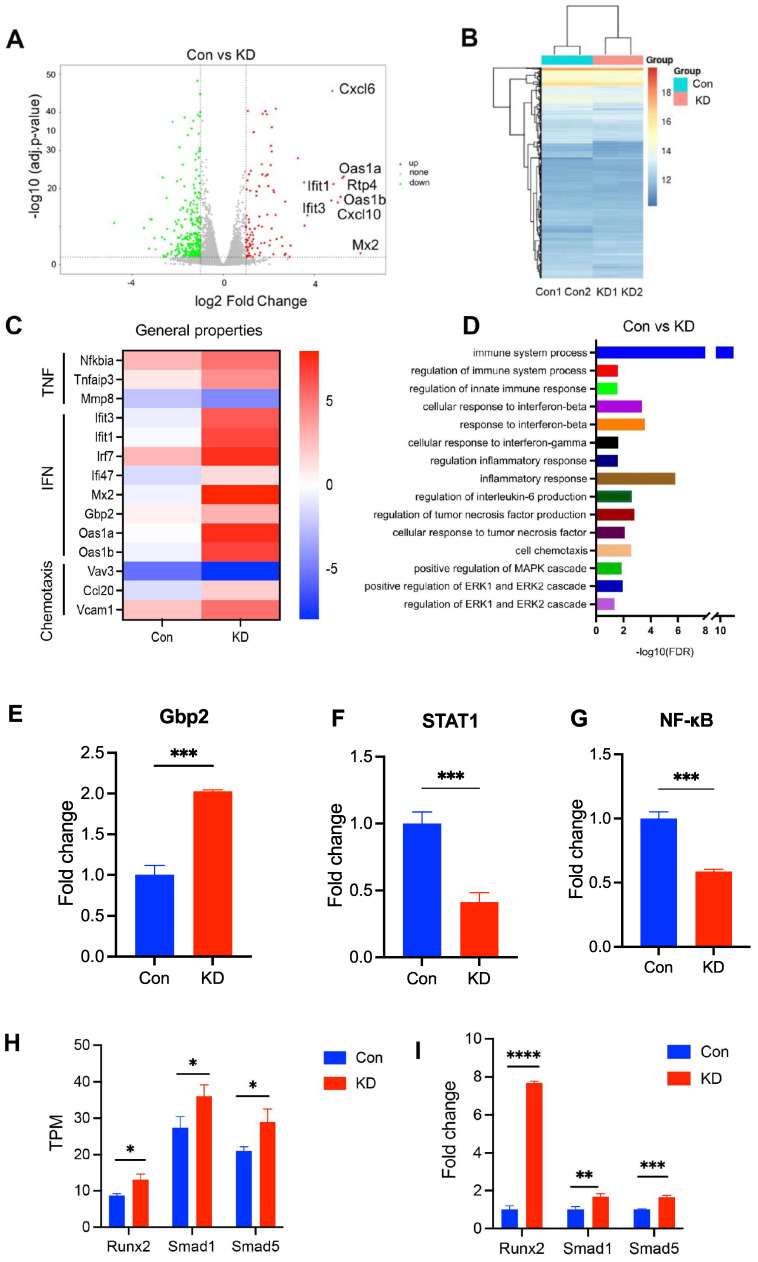
** RNA-seq analysis of NLRP3-modulated genes**. **(A),** A volcano plot illustrating differentially regulated gene expression from RNA-seq analysis between the negative control siRNA- (Con) and NLRP3 siRNA-treated osteoblasts (NLRP3 knock-down group, KD). Genes up-regulated and down-regulated are shown in red and green, respectively (The top 10 up-regulated categories are shown). Values are presented as the log2 of tag counts. **(B),** Heat map of RNA-seq expression data showing the genes that were differentially regulated following NLRP3 knock-down. **(C)**, Heatmap of representative TNF, interferon (IFN), cell chemotaxis-related genes. **(D)**, Gene ontology (GO) functional clustering of genes that were different expressed for biological processes. **(E-G)** Relative expression levels of Gbp2 (E), STAT1 (F) and NF-κB (G) obtained by qPCR. **(H-I)**, Validation of RNA-Seq gene Runx2, Smad1, Smad5 expression by qPCR. TPM values obtained by RNA-Seq analysis (H) and relative expression levels obtained by qPCR (I). Data are represented as means ± SD. Statistical significance was calculated by Student t-test (Compared with Con, *P < 0.05, **P < 0.01, ***P < 0.001, ****P < 0.0001).

We next investigated the effect of NLRP3 on the pathology of PMOP. To address this, we carried out gene expression profiling using RNA-seq from the OVX rat derived osteoblasts to identify transcriptomic changes by NLRP3 siRNA treatment. We first evaluated the knockdown efficacy of several NLPR3 siRNA. All three NLPR3 siRNA significantly decreased NLRP3 levels, and NLPR3 siRNA-3 had the highest knockdown efficiency by 89% (Figure S1). The NLPR3 siRNA-3 was used for subsequent experiments. Then we identified a total of 275 genes that fulfilled our criteria (fold change > 1.5 and q-value < 0.05) as DEGs (Figure 2A-B). Of the top ten up-regulated genes, nine genes (except Mx2) were enriched in immune system process, and five genes (lfit1, lfit3, Oas1a, Oas1b, Mx2) were related to interferon signaling pathway (Figure 2A, 2C), indicating that immune system and interferon signaling pathway may play an important role in NLRP3-mediated PMOP. Moreover, other interferon signal pathway-related genes Irf7, Ili47, Gbp2 were also up-regulated. Some TNF-linked genes, such as Nfkbia, Tnfaip3, Mmp8 were significantly changed in NLRP3 siRNA treated-osteoblasts compared with the control. Some genes, such as Vav3, Ccl20, Vcam1, with functions related to cell chemotaxis were significantly changed as well (Figure 2C). GO biological process analysis revealed that these DEGs have functions related to immune system process, regulation of innate immune response, response to interferon-beta, cellular response to interferon-gamma, regulation of inflammatory response, cellular response to tumor necrosis factor, regulation of interleukin-6 production, cell chemotaxis, positive regulation of MAPK, ERK1 and ERK2 cascade (Figure 2D). To further verify that reduction of NLRP3 expression in osteoblasts regulates immune system process and interferon signal pathway, we analyzed the levels of immune system process- and interferon-related genes Gbp2, STAT1 and NF-κB. The results showed that compared with Con, NLRP3 knockdown group had higher Gbp2 levels but lower STAT1 and NF-κB levels (Figure 2E-G). These may be associated with IL-18, which is regulated by NLRP3, can activate NF-κB, and then impact the level of interferon-related gene STAT1 26, 27. To determine whether knockdown of NLRP3 promotes osteogenesis, we analyzed the TPM values of osteogenesis-related genes Runx2, Smad1, Smad5. RNA sequencing data showed that the TPM values of Runx2, Smad1, and Smad5 were significantly increased in osteoblasts treated with NLRP3 siRNA (Figure 2H) and these results were further confirmed by qPCR (Figure 2I).

### The characterization of CH6-LNPs-siNLRP3

**Figure 3:**
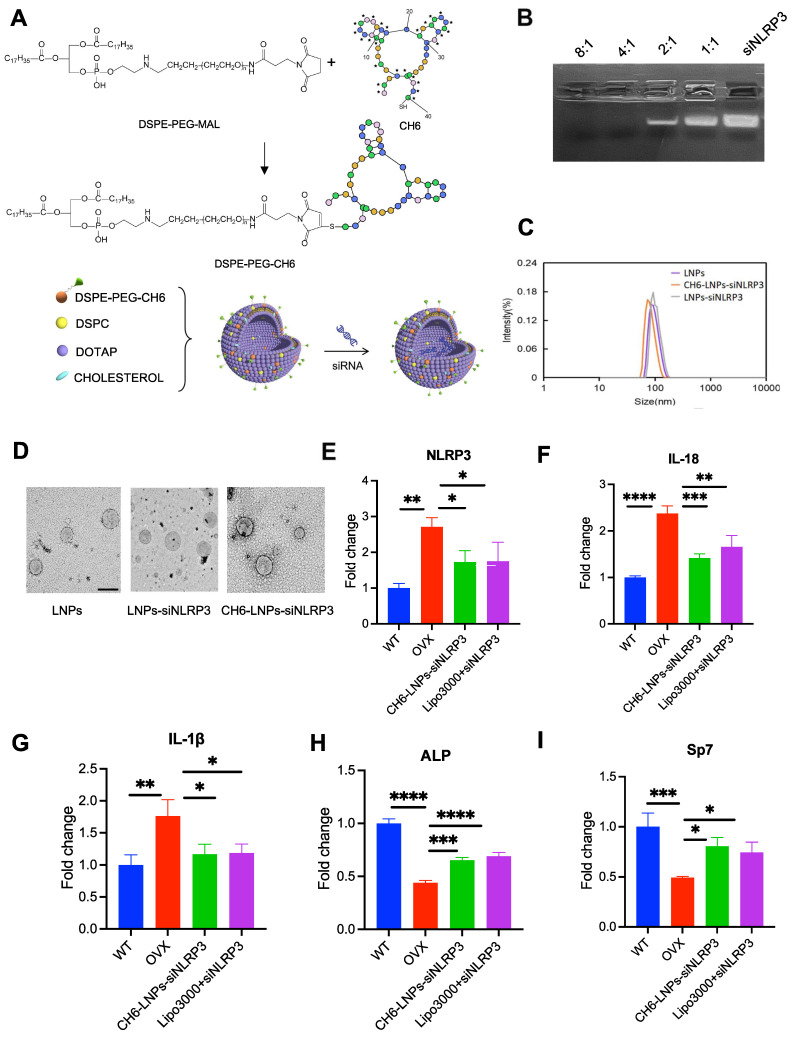
** Characterization of the prepared lipid nanoparticles. (A)**, The Schematic of bone-targeted-LNPs used in present study. The designed LNPs, termed as CH6-LNPs-siNLRP3, displays bone-targeted adaptor (CH6) on the surface, and contains the NLRP3 siRNA (siNLRP3). **(B)**, The N/P ratio (a molar ratio) of LNPs-siNLRP3 measured by gel retardation assay. **(C)**, Representative size distributions of LNPs, LNPs-siNLRP3 and CH6-LNPs-siNLRP3 measured by dynamic light scattering method.** (D)**, Negative-stained TEM image of CH6-LNPs-siNLRP3. The samples were applied to copper grids, negatively stained and imaged by Hitachi TEM at 100 KV at 60,000× magnification. Scale bar represents 100 nm. **(E),** The knockdown efficiency of CH6-LNPs-siNLRP3 was tested by qPCR. Osteoblasts isolated from OVX rats were plated at 70% confluence and transfected with CH6-LNPs-siNLRP3 and siNLRP3 carried by lipo3000. Osteoblasts from non-OVX (WT) and OVX rats treated with negative control siRNA were used as controls. The mRNA levels of IL-18 **(F)**, IL-1β **(G)**, Sp7 **(H)**, and ALP **(I)** in CH6-LNPs-siNLRP3-treated osteoblasts isolated from OVX rats were detected by qPCR. Data are represented as means ± SD. Statistical significance was calculated by Student t-test (Compared with OVX, *P < 0.05, **P < 0.01, ***P < 0.001, ****P < 0.0001).

Next, we investigated the effect of NLRP3 reduction on OVX rats. We prepared CH6-LNPs loaded with NLRP3 siRNA. CH6 was conjugated on the surface of LNPs for the specific delivery of siRNA to osteoblasts 22 (Figure 3A). Firstly, we optimized the N/P ratio of LNPs. The results showed that when the N/P ratio was 4:1, siRNA was completely encapsulated into LNPs (Figure 3B). The optimal N/P ratio of 4:1 was used for subsequent experiments. Then CH6 was conjugated on the surface of LNPs (Figure S2) and the grafted amount of CH6 on LNPs was 153.22 µg/ml by NanoDrop 2000c spectrophotometer.

DLS results showed that the average hydrodynamic diameters of LNPs, LNPs-siNLRP3 and CH6-LNPs-siNLRP3 were 89.44 ± 12.31 nm, 92.60 ± 13.28 nm and 96.64 ± 16.83 nm, and they had a narrow particle diameter distribution with a polydispersity index (PDI) of 0.19 ± 0.03, 0.21 ± 0.04 and 0.22 ± 0.03, respectively (Figure 3C, Table 2). The zeta potentials of LNPs, LNPs-siNLRP3 and CH6-LNPs-siNLRP3 were 44.67 ± 0.25 mV, 39.87 ± 1.60 mV and 38.37 ± 1.86 mV, respectively (Table 2). Our TEM results demonstrated that LNPs, LNPs-siNLRP3 and CH6-LNPs-siNLRP3 formed nanoparticles (Figure 3D). Moderate LNP sizes (70-200 nm) have been reported to have extended circulation time 28. Thus, our CH6-LNPs-siNLRP3 with an average size of 96.6 nm seems to be optimal for bone-targeting application since their size may favor extended circulation within the bloodstream. We also investigated the release behavior of CH6-LNPs-siNLRP3, as shown in Figure S3, 97.5% of siNLRP3 was released from CH6-LNPs-siNLRP3 after 96 h incubation with PBS (Figure S3).

Then we examined the NLRP3 knockdown efficiency of CH6-LNPs-siNLRP3 and siNLRP3 in lipo3000 (Lipo3000+siNLRP3) by qPCR, and osteoblasts from WT rats were used as a control. The results showed that compared with osteoblasts from WT rats, higher levels of NLRP3 were observed in osteoblasts from OVX rats (Figure 3E). However, after CH6-LNPs-siNLRP3 and Lipo3000+siNLRP3 treatment, the expression of NLRP3 were significantly reduced (Figure 3E). These results were further confirmed by Western blot (Figure S4). Consistently, the levels of pro-inflammatory cytokines IL-1β and IL-18 were decreased in osteoblasts from OVX rats treated with CH6-LNPs-siNLRP3 or Lipo3000+siNLRP3 (Figure 3F-G). Interestingly, bone formation markers ALP and Sp7 were significantly increased in osteoblasts treated with CH6-LNPs-siNLRP3 and Lipo3000+siNLRP3 (Figure 3H-I). Furthermore, differentiation of osteoblasts was assessed by Alizarin red S and ALP staining. As shown in Figure S5, compared with osteoblasts from WT, osteoblasts from OVX rats had lower levels of ALP activity and matrix mineralization. However, CH6-LNPs-siNLRP3 and Lipo3000+siNLRP3 treatment significantly increased the ALP activity (Figure S5A-B). Consistently, matrix mineralization was enhanced by CH6-LNPs-siNLRP3 and Lipo3000+siNLRP3 treatment (Figure S5A, C). The above results indicated that CH6-LNPs-siNLRP3 stimulates osteoblast differentiation by activating ALP and enhancing osteoblast matrix mineralization.

### CH6-LNPs-siNLRP3 has no obvious cytotoxicity

We evaluated the cytotoxicity of these nanoparticles in osteoblasts and 293T cells. The MTT assay revealed that no obvious cytotoxicity was observed in osteoblasts and 293T cells treated with LNPs, LNPs-siNLRP3 and CH6-LNPs-siNLRP3, even though the concentration of the particles was increased to 0.2 mg/ml (Figure S6). To investigate the effect of CH6-LNPs-siNLRP3 on the toxicity to rats, CH6-LNPs-siNLRP3 was injected to rats via tail vein, and no obvious toxicity was observed in the liver and kidneys of rats treated with or without CH6-LNPs-siNLRP3 (Figure S7).

### CH6-LNPs-siNLRP3 selectively delivers siRNA to bone tissue

**Figure 4:**
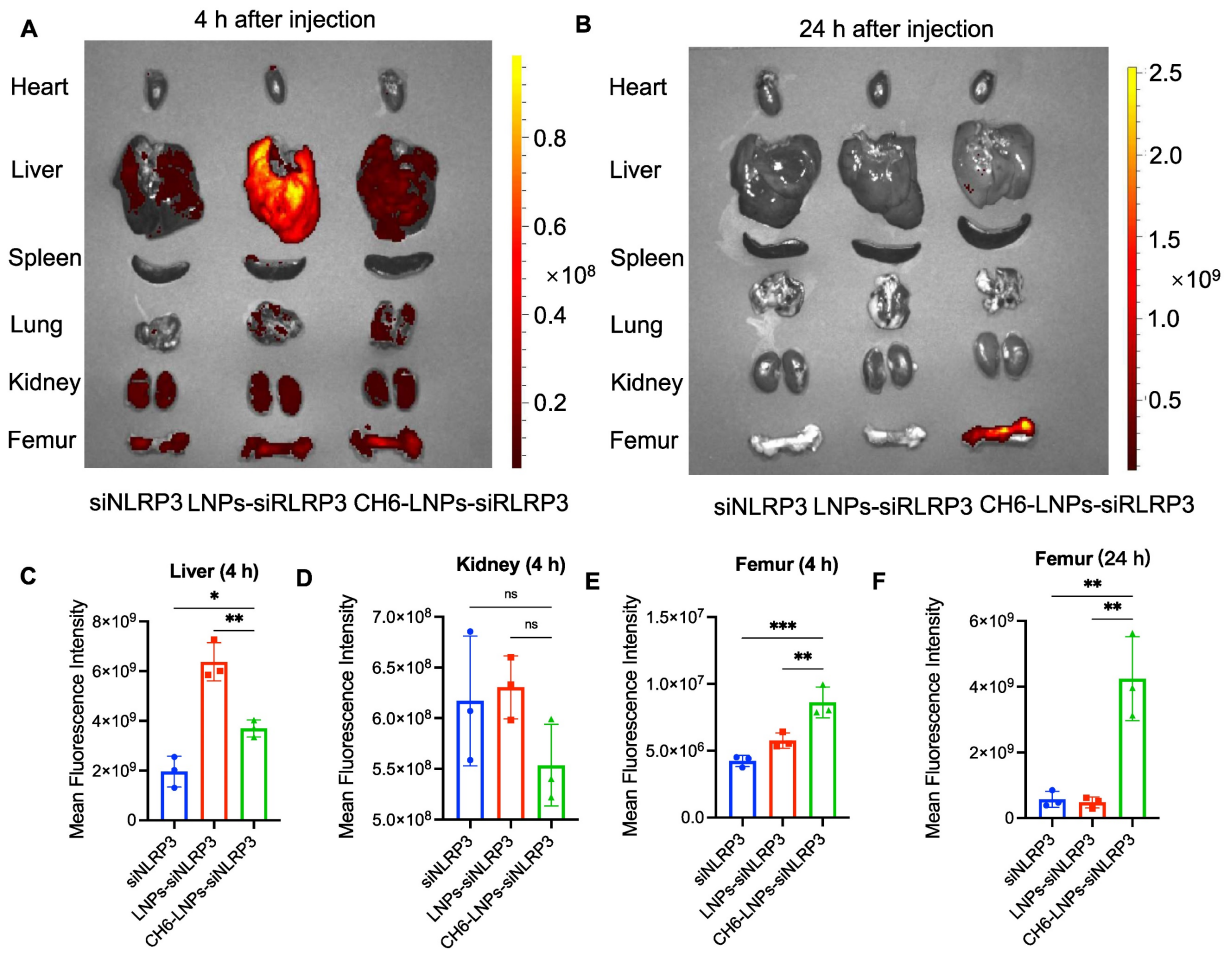
** Tissue distribution of CH6-LNPs-siNLRP3 *in vivo*.** The rats were administered with siNLRP3, LNPs-siNLRP3 and CH6-LNPs-siNLRP3 containing Cy3-labeled siRNA via tail vein injection and the localization of Cy3-labeled siRNA was visualized by biophotonic imaging 4 h **(A)** and 24 h** (B)** post injection. Quantitative analysis of siRNA in livers (4 h) **(C)**, kidneys (4 h) **(D)**, Femurs (4 h) **(E)** and Femurs (24 h)** (F)** from rats injected with siNLRP3, LNPs-siNLRP3 and CH6-LNPs-siNLRP3 by Living Image®4.4, respectively. n = 3 per group. Results are shown as mean ± SD. For **(C-F)**, Statistical significance was calculated by one-way ANOVA followed by Dunnett's test (Compared with CH6-LNPs-siNLRP3, *P < 0.05, ns, not significant).

To confirm the specific targeting of CH6 to bone tissue, we examined the tissue distribution of LNPs containing Cy3-labeled siNLRP3 by biophotonic imaging technology. Fluorescence signal intensity of CH6-LNPs-siNLRP3 in bone was significantly higher, whereas the fluorescence signal intensities in liver and kidney were lower compared to that of LNPs-siNLRP3 at 4 h after a tail vein injection, the differences did not reach statistical significance in the kidney (Figure 4A, C-E). Almost no fluorescence intensity in the liver, kidney was observed in all administration groups, while some fluorescence intensity was still observed in the bones of CH6-LNPs-siNLRP3-treated group 24 h after injection (Figure 4B, F). Consistent with previous report 22, there were very low fluorescence signals in the hearts, lungs and spleens in all the administration groups (Figure 4A-B).

### CH6-LNPs-siNLRP3 promotes bone formation in OVX rat model of PMOP

**Figure 5:**
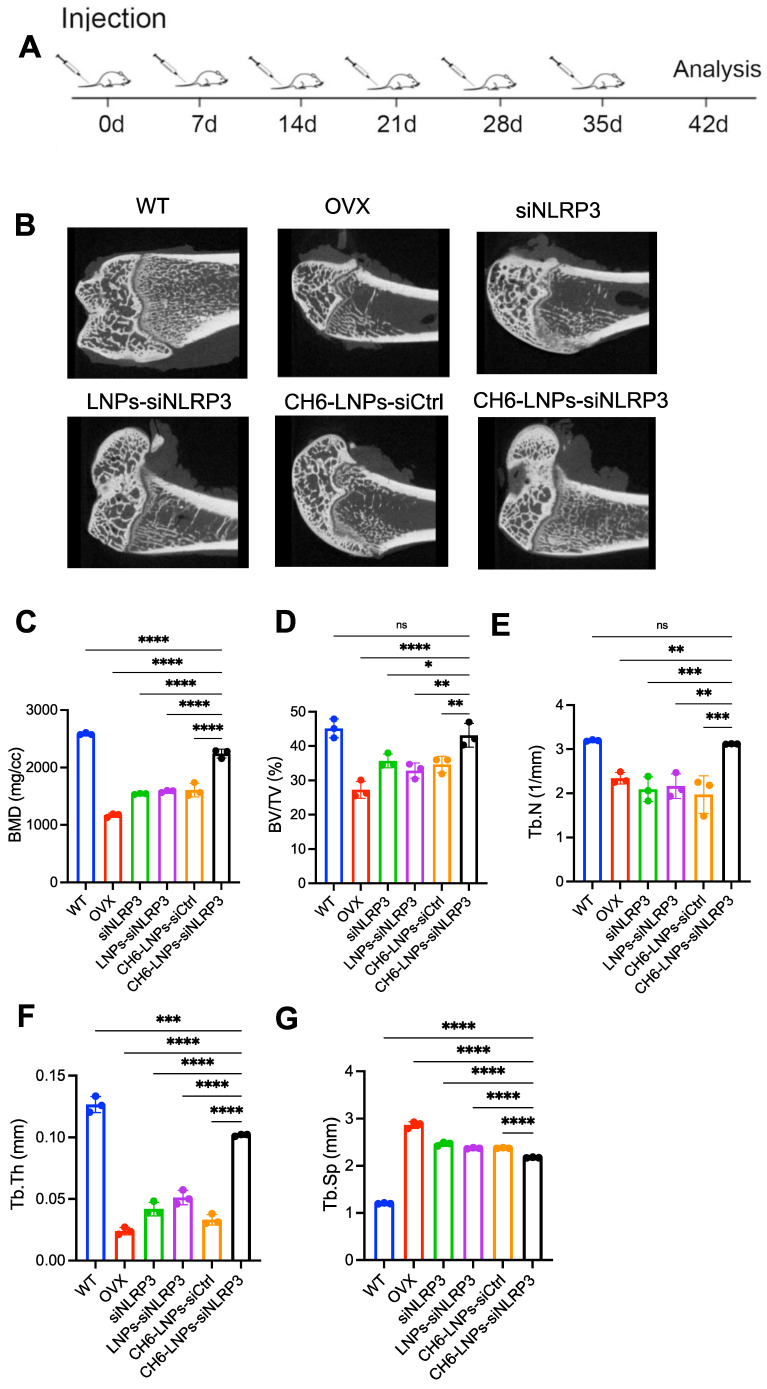
** CH6-LNPs-siNLRP3 treatment improve MicroCT parameters. (A)**, schematic diagram illustrating the experimental procedure. **(B)**, Representative 3D microarchitecture of the femurs in each group, obtained by *in vivo* microCT. BMD **(C)**, BV/TV **(D)** and three-dimensional architecture parameters, Tb.N **(E)**, Tb.Th **(F)**, and Tb.Sp **(G)** in trabecular bone from the OVX rats treated with PBS, NLRP3 siRNA, LNPs-siNLRP3, CH6-LNPs-siNLRP3 and CH6-LNPs-siCtrl were detected by MicroCT. n = 3 per group. Results are shown as mean ± SD. For **(C-G)**, Statistical significance was calculated by one-way ANOVA followed by Dunnett's test (Compared with CH6-LNPs-siNLRP3, *P < 0.05, **P < 0.01, ***P < 0.001, ****P < 0.0001, ns, not significant).

We performed hemolysis assay to investigate the biocompatibility of the CH6-LNPs-siNLRP3. 2, 10, 50, 100, 200 or 400 μg/ml of CH6-LNPs-siNLRP3 was incubated with 2% red blood cells suspension at room temperature for 4 h. The results showed the hemolytic ratio was below 5% in all the test concentration (Figure S8), indicating that CH6-LNPs-siNLRP3 was biocompatible. Then we investigated the effect of CH6-LNPs-siNLRP3 on the OVX rat model of PMOP. The OVX rat model is an approved preclinical model by the Food and Drug Administration (FDA) for studying how the decline in endogenous estrogen generation by the ovaries at menopause results in PMOP and how potential interventions can maintain bone metabolism in this state 29. After OVX operations, we randomly selected six rats and then tested their E2 levels, the results showed that E2 levels were significantly decreased compared with WT (Figure S9). We further performed the micro-CT of the left femur of rats, the results showed that BMD was significantly decreased in OVX rats compared with that in WT rats (Figure S10), the above results indicated the successful construction of the osteoporotic rat models. Six periodic injections of NLRP3 siRNA (1.0 mg/kg) encapsulated with or without LNPs were administered to OVX rats at an interval of one week and microCT scanning were carried out one week after the last treatment (Figure 5A). After treatment, higher bone mass and better-organized microarchitecture were found in trabecular bone from the OVX rats treated with CH6-LNPs-siNLRP3 relative to PBS-, siNLRP3-, LNPs-siNLRP3- and CH6-LNPs-siCtrl-treated groups (Figure 5B). Consistently, all the microCT parameters, including BMD, BV/TV, Tb.N, Tb.Th, and Tb.Sp were significantly improved in the CH6-LNPs-siNLRP3-treated group relative to other control groups (Figure 5C-G).

**Figure 6:**
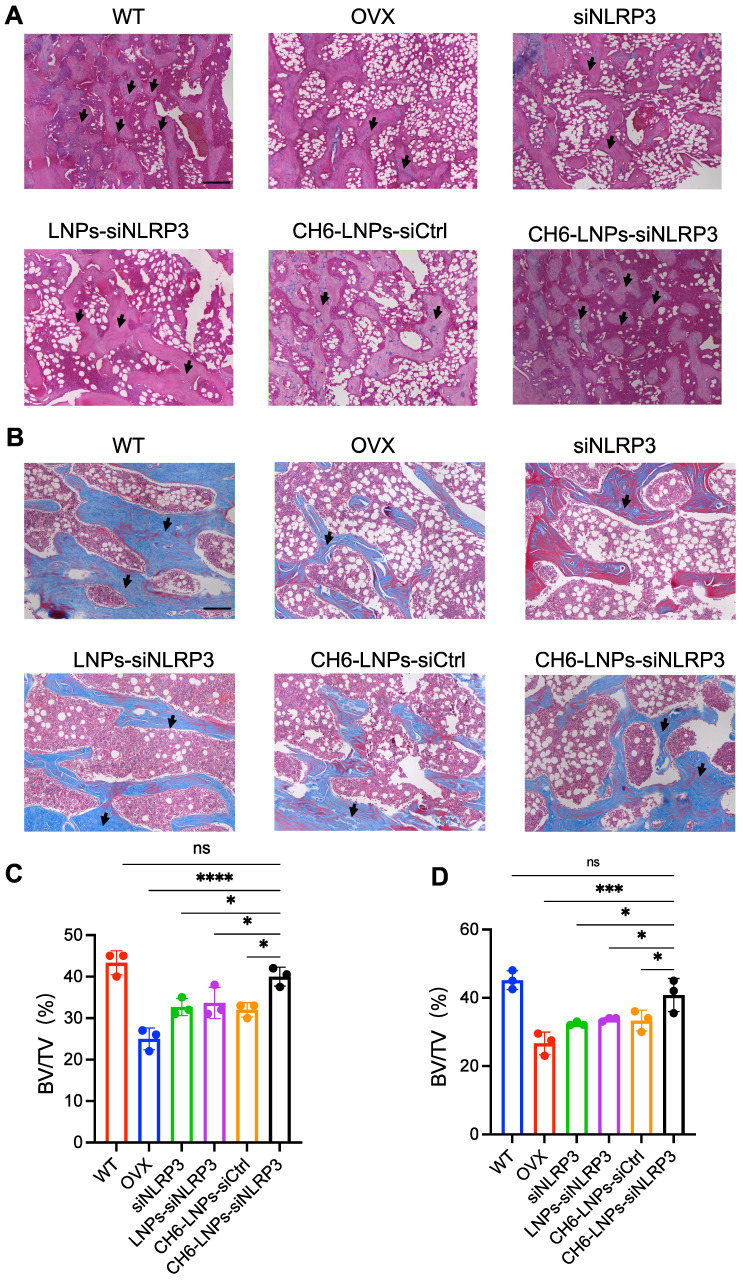
** CH6-LNPs-siNLRP3 increases bone formation in OVX rats.** (A-B), Representative images of HE staining (A) and Masson staining (B) of decalcified bone sections. Scale bar represents 200 μm. (C-D), BV/TV in tissue sections of HE staining (C) and Masson staining (D) were quantified by BIOQUANT OSTEO software (n = 3 per group). Results are shown as mean ± SD. Statistical significance was calculated by one-way ANOVA followed by Dunnett's test (Compared with CH6-LNPs-siNLRP3, *P < 0.05, ***P < 0.001, ****P < 0.0001, ns, not significant).

**Figure 7:**
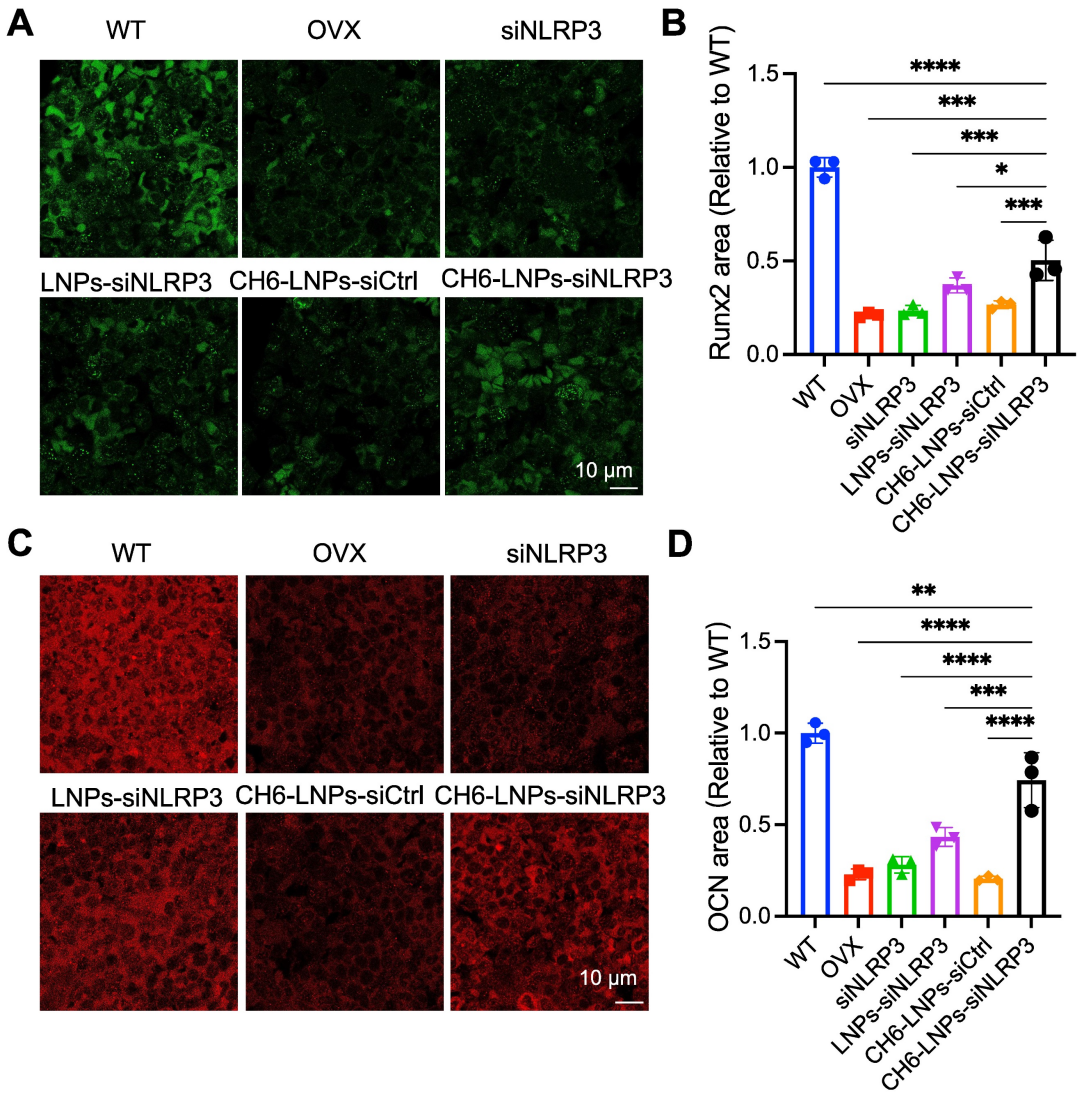
** CH6-LNPs-siNLRP3 enhances osteogenesis. (A, C)** Representative confocal images of Runx2 (A), OCN (C) in the decalcified bone sections of OVX rats treated with PBS, NLRP3 siRNA, LNPs-siNLRP3, CH6-LNPs-siNLRP3 and CH6-LNPs-siCtrl. Scale bar represents 10 μm. **(B, D)** The fluorescent area of Runx2 (B) in (A) and OCN (D) in (C) was quantified by Image J software. n = 3 per group. Statistical significance was calculated by one-way ANOVA followed by Dunnett's test (Compared with CH6-LNPs-siNLRP3, *P < 0.05, **P < 0.01, ***P < 0.001, ****P < 0.0001).

HE staining and Masson staining were carried out to evaluate bone formation of OVX rats. HE staining showed that compared with control, CH6-LNPs-siNLRP3 had higher BV/TV, suggesting that CH6-LNPs-siNLRP3 promoted the bone formation of OVX rats (Figure 6A, C). Masson staining further verified that CH6-LNPs-siNLRP3 increased bone formation (Figure 6B, D). Interestingly, a significant reduction in the production of fat droplets can be seen in the CH6-LNPs-siNLRP3-treated group compared to the PBS-treated control group (Figure 6A, C). Moreover, we detected the levels of osteogenic markers OCN and Runx2 by immunofluorescence. Higher levels of Runx2 were found in CH6-LNPs-siNLRP3-treated group relative to PBS-, siNLRP3-, LNPs-siNLRP3- and CH6-LNPs-siCtrl-treated groups (Figure 7A-B). Consistently, significantly increased OCN levels were observed in CH6-LNPs-siNLRP3-treated group (Figure 7C-D). The above results indicating that CH6-LNPs-siNLRP3 promoted osteogenesis of OVX rats.

### CH6-LNPs-siNLRP3 reduces inflammation in OVX rats

**Figure 8:**
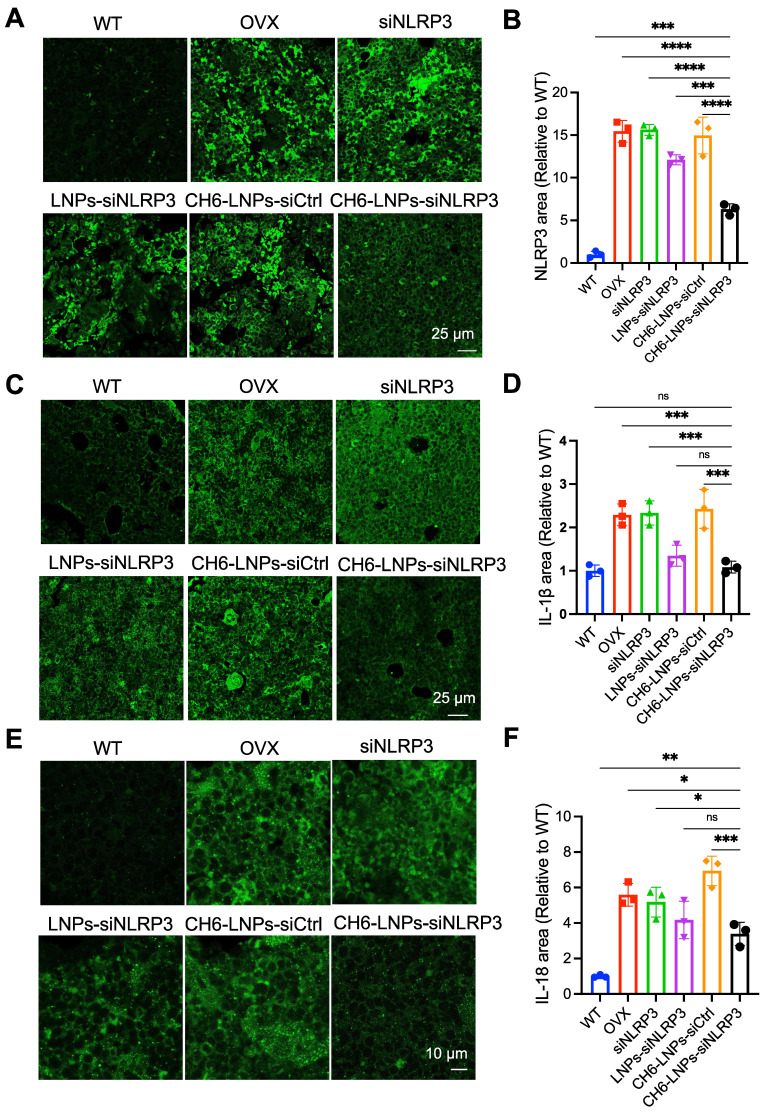
** CH6-LNPs-siNLRP3 reduces inflammation in OVX rats. (A, C, E)** Representative confocal images of NLRP3 (A), IL-1β (C), IL-18 (E) in the decalcified bone sections of OVX rats treated with PBS, NLRP3 siRNA, LNPs-siNLRP3, CH6-LNPs-siNLRP3 and CH6-LNPs-siCtrl. Scale bar represents 10 μm or 25 μm. **(B, D, F)** The fluorescent area of NLRP3 (B) in (A), IL-1β (D) in (C), and IL-18 (F) in (E) was quantified by Image J software. n = 3 per group. Statistical significance was calculated by one-way ANOVA followed by Dunnett's test (Compared with CH6-LNPs-siNLRP3, *P < 0.05, **P < 0.01, ***P < 0.001, ****P < 0.0001, ns, not significant).

The NLRP3 inflammasome mediates the activation of inflammatory IL-1β, and IL-18, causing inflammation and inducing inflammatory cell death. IF assays were carried out to assess the levels of NLRP3, IL-1β and IL-18 in OVX rats. The results showed that the levels of NLRP3 were significantly reduced in CH6-LNPs-siNLRP3-treated group compared with that in PBS-, siNLRP3-, LNPs-siNLRP3- and CH6-LNPs-siCtrl-treated groups (Figure 8A-B). Consistently, significantly reduced IL-18 and IL-1β levels were observed in CH6-LNPs-siNLRP3-treated group (Figure 8C-F).

## Discussion

Previous reports have demonstrated that NLRP3 inflammasome is critical in the development and progression of many metabolic diseases driven by chronic inflammation, including Alzheimer's disease, and diabetes 30, 31, and NLRP3 may contribute to bone loss caused by bacterial infection 10, but the effect of NLRP3 inflammasome on the pathology of PMOP remains poorly understood. In this study, we first found that the levels of NLRP3 were increased in patients with PMOP, and negatively correlated with E2 levels, which is consistent with our previous report that NLRP3 were highly expressed in OVX mice 32. Estrogen deficiency may induce chronic inflammation, increase the levels of NF-κB pathway, then upregulate NLRP3 expression 33. The above results indicated that NLRP3 inflammasome may contribute to the pathology of PMOP and may be a new biomarker for PMOP diagnosis. We further investigated the mechanisms underlying the effect of NLRP3 inflammasome on osteoblasts by RNA-seq. Our results showed that reduction of NLRP3 expression influenced signal pathways including immune system process, cellular response to tumor necrosis factor, and response to interferon-beta, this may be associated with that NLRP3 inflammasome is a vital component of the natural immune system 34. The immune system plays a key role in bone tissue formation and bone resorption 35.

The NLRP3 inflammasome involved in the activation of inflammatory responses by promoting the maturation and release of pro-inflammatory cytokines, such as IL-1β and IL-18. IL-1β, a key member of the IL-1 family, contributes significantly to bone loss after estrogen deficiency 36, 37. A previous report showed high doses of IL-1β impede osteogenic differentiation through the activation of NF-kB, thereby suppressing the bone morphogenetic protein (BMP)/Smad signaling pathway 38. Another study reported that IL-1β reduces Runx2 activation and hinders osteoblastic differentiation by triggering the MAPK pathway 39. Consistent with the above reports, our results showed that reduction of NLRP3 decreased the levels of IL-1β and IL-18, enhanced the levels of Runx2, OCN, ALP, Sp7, Smad1 and Smad5, therefore promoted bone formation.

Previous reports showed that the interferon signal pathway plays an inhibitory role in osteoclast generation 40-42. Administration of interferon-beta prevented bone loss in rats with ovariectomy-induced osteoporosis 40. Interestingly, interferon signal pathway-related genes lfit1, lfit3, Oas1a, Oas1b, Mx2 were of the top ten up-regulated genes in NLRP3 siRNA-treated osteoblasts, suggesting that interferon signal pathway may play an important role in NLRP3 siRNA attenuating osteoporosis. Notably, Tnfaip3 was significantly up-regulated in osteoblasts with NLRP3 knock-down, which is consistent with the report that Tnfaip3 acted as a negative regulator of the NLRP3 inflammasome 43.

Molecular therapy via siRNA has proved to have high therapeutic potential for many diseases 44, 45. However, RNA agents are difficult to reach the target organs or tissues, unstable and easy to be degraded. To protect NLRP3 siRNA from hydrolysis and specially deliver it to bone, we here used LNPs as our nanocarrier, which are highly biocompatible and have been approved by FDA for drug delivery. There are two major types of bone-targeting molecules including molecules selective for bone-related cells and those with high affinity for the inorganic matrix of the bone. The latter, such as oligopeptides and bisphosphonates are conventional bone-targeting ligands 46, 47, Novel targeting strategies apply nucleic acid-based aptamers, antibodies or protein-based ligands to precisely deliver genes or drugs to certain bone cell subtypes 48. Aptamers can be easily synthesized and modified for high self-stability, rapid tissue penetrability, and low immunogenicity, showing promising application potential for a targeted drug delivery system. In this study, osteoblast-specific aptamer CH6 was loaded on the surface of LNPs to specifically deliver siNLRP3 to bone. Our *in vivo* experiment results consistently showed that CH6 facilitated the delivery of siNLRP3 to bone tissue. Moreover, CH6-LNPs-siNLRP3 significantly promoted bone formation, enhanced bone mass, improved bone microarchitecture and increased mechanical strength compared with LNPs-siNLRP3.

A nanoparticle should fulfill the following criteria to achieve the desired clinical application: high biocompatibility, low cytotoxicity, immunogenicity and side effect, sufficient loading capacity, and easy modification for special applications. In this study, CH6-LNPs-siNLRP3 also did not show obvious toxicity *in vivo* and *ex vivo*. Our present results indicate that CH6-LNPs-siNLRP3 has potential application for the treatment of PMOP.

## Conclusion

In summary, we found that the levels of NLRP3 inflammasome were significantly increased in patients with PMOP, and were negatively correlated with estradiol variables. Reduction of NLRP3 expression influenced signal pathways including immune system process, cellular response to tumor necrosis factor, and response to interferon-beta. Notably, immune system process and interferon signal pathway-related genes were of the top ten up-regulated genes in NLRP3 siRNA-treated osteoblasts. Then we confirmed that CH6-LNPs-siNLRP3 selectively delivered siRNA to bone tissue, and promoted bone formation and bone mass, improved bone microarchitecture and mechanical properties of OVX rats by decreasing the levels of NLRP3, IL-1β and IL-18 and increasing the levels of OCN and Runx2. These results indicate that NLRP3 inflammasome plays a key role in the pathology of PMOP and has potential application for the treatment of PMOP.

## Supplementary Material

Supplementary figures and table.

## Figures and Tables

**Table 1:** Primers

rNLRP3	5'- GGGACTCAAGCTCCTCTGTG -3'
	5'- GAGGCTCTGGTTATGGGTCA -3'
rIL-18	5'- GACATCCTTCCATCCTTCACAGATAG -3'
	5'- GACCGAACAGCCAACGAATCC -3'
rIL-1β	5'- TTTGTCGTTGCTTGTCTCTCCTTG -3'
	5'- ATGCCTCGTGCTGTCTGACC -3'
rGbp2	5'-AGCAGCACCTTCGTCTACAACAG-3'
	5'-AGCCCACAAAGTTAGCAGAGTCG-3'
rSTAT1	5'-TCGCACCTTCGTCCTCTTCCAG-3'
	5'-TTCACCAACAGTCTCAGCTTCACAG-3'
rNF-κB	5'-TGTGGTGGAGGACTTGCTGAGG-3'
	5'-AGTGCTGCCTTGCTGTTCTTGAG-3'
rGAPDH	5'-CAAGTTCAACGGCACAGTCA-3'
	5'-CCATTTGATGTTAGCGGGAT-3'
rRunx2	5'-CCCAACT-TCCTGTGCTCC-3'
	5'-AGTGAAACTCTTGCCTCGTC-3'
rALP	5'-TGGACGGTGAACGGGAGAAC-3'
	5'-GGACGCCGTGAAGCAGGTGA-3'
rSp7	5'-CTTCTCAAGCACCAATGGA-3'
	5'-CTAGGCAGGCAGTCAGAA-3'
rSmad1	5'-CCGCCTCTTACCTGCCTCCTGAA-3'
	5'-GAACGCTTCGCCCACACGGTT-3'
rSmad5	5'-GCTTCTGGCTCAGTCAGTCAACC-3'
	5'-ATCCTGTCGGTGGTACTCTGCTC-3'

**Table 2:** Size, PDI and zeta potential of CH6-LNPs-siNLRP3

	DLS (nm)	PDI	Zeta (mV)
LNPs	89.44 ± 12.31	0.19 ± 0.03	44.67 ± 0.25
LNPs-siNLRP3	92.60 ± 13.28	0.21 ± 0.04	39.87 ± 1.60
CH6-LNPs-siNLRP3	96.64 ± 16.38	0.22 ± 0.03	38.37 ± 1.86

## Abbreviations

PMOP: postmenopausal osteoporosis
OVX: ovariectomized
TNF-α: tumor necrosis factor alpha
IL-6: interleukin-6
MMP-3: matrix metalloproteinase-3
NF-κB: nuclear factor kappa B
LNPs: lipid nanoparticles
E2: estradiol
RNA-seq: RNA sequencing
DEGs: differentially expressed genes
DLS: dynamic light scattering
PDI: polydispersity index
TEM: Transmission Electron Microscopy
microCT: microcomputed tomography
BMD: bone mineral density
BV/TV: relative bone volume
Tb.N: trabecular number
Tb.Th: trabecular thickness
Tb. Sp: trabecular spacing
SMI: structure model index
FDA: Food and Drug Administration
