# Supplementary material for: Osteoblast-specific down-regulation of NLRP3 inflammasome by aptamer-functionalized liposome nanoparticles improves bone quality in postmenopausal osteoporosis rats
Source: Theranostics. 2024 Jun 24;14(10):3945–62. doi: 10.7150/thno.95423 (PMC11234270; doi:10.7150/thno.95423)
Supplement: Supplementary file 1 — Supplementary figures and table. [file thnov14p3945s1.pdf]

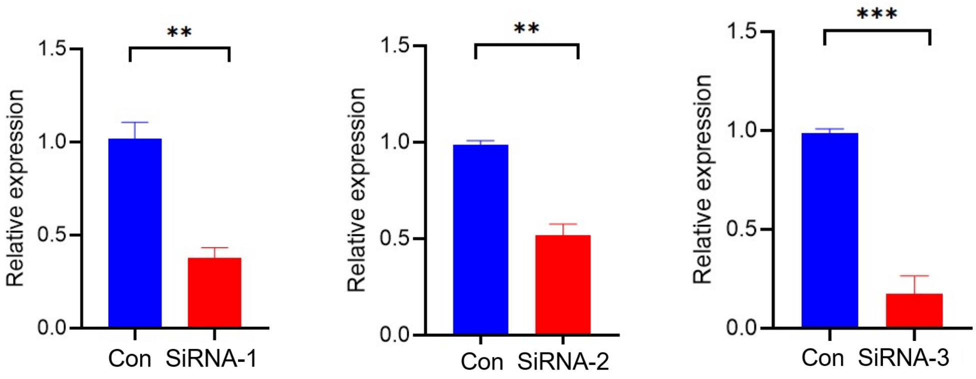

Figure S1. The knockdown efficiency of NLRP3 siRNA tested by qPCR. Osteoblasts isolated from rats were plated at 70% confluence and transfected with NLRP3 siRNA-1, siRNA-2 and siRNA-3 using lipofectamine 3000, respectively, then NLRP3 mRNA levels were detected by qPCR. Statistical significance was calculated by Student t-test (Compared with Con, $^{**}P < 0.01$, $^{***}P < 0.001$)

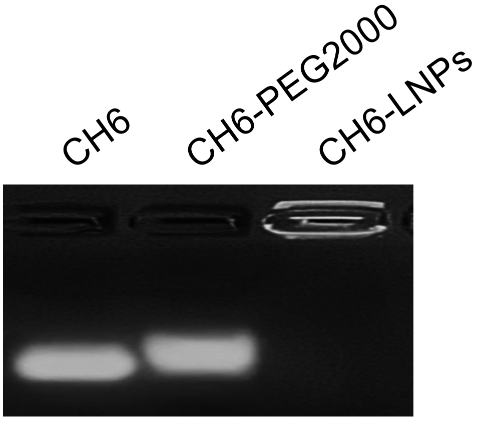

Figure S2. The conjugation of CH6 to the surface of LNPs was analyzed by agarose gel. CH6, CH6-PEG2000 and CH6-LNPs were applied to 3% agarose gel, and stained by ethidium bromide.

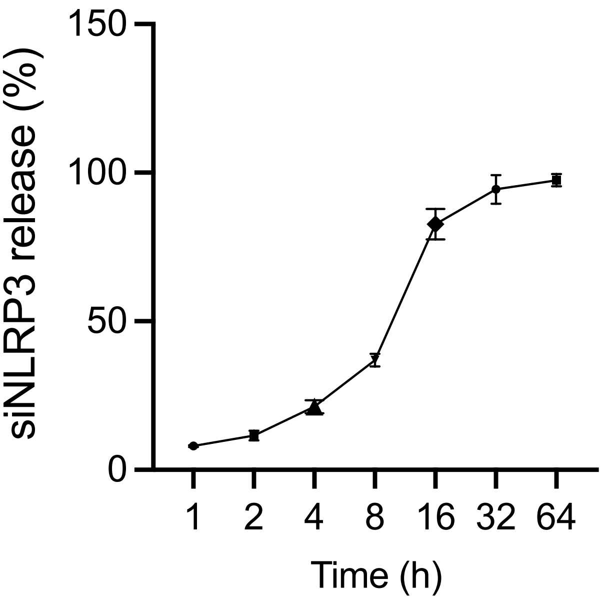

Figure S3. The release kinetics of siNLRP3 from CH6-LNPs-siNLRP3. CH6-LNPs loaded with Cy3-labeled siNLRP3 were incubated with pH 6.5 PBS for 1 h, 2 h, 4 h, 8 h, 16 h, 32 h, 64 h at 37 °C. The released siNLRP3 were measured using a fluorescence assay.

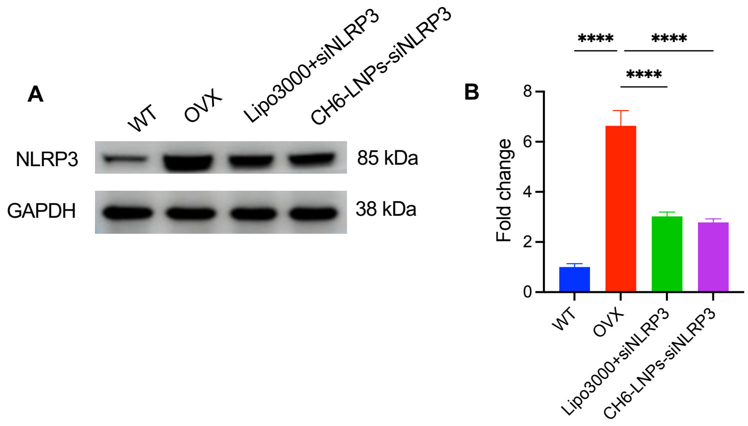

Figure S4. **The knockdown efficiency of CH6-LNPs-siNLRP3 was tested by Western blot.** **(A)** Representative images of NLRP3 in osteoblasts isolated from OVX rats transfected with CH6-LNPs-siNLRP3 and siNLRP3 in lipo3000. **(B)** Densitometry analysis of NLRP3 was quantified by Image J. Statistical significance was calculated by one-way ANOVA followed by Dunnett's test (Compared with OVX, ****$P < 0.0001$)

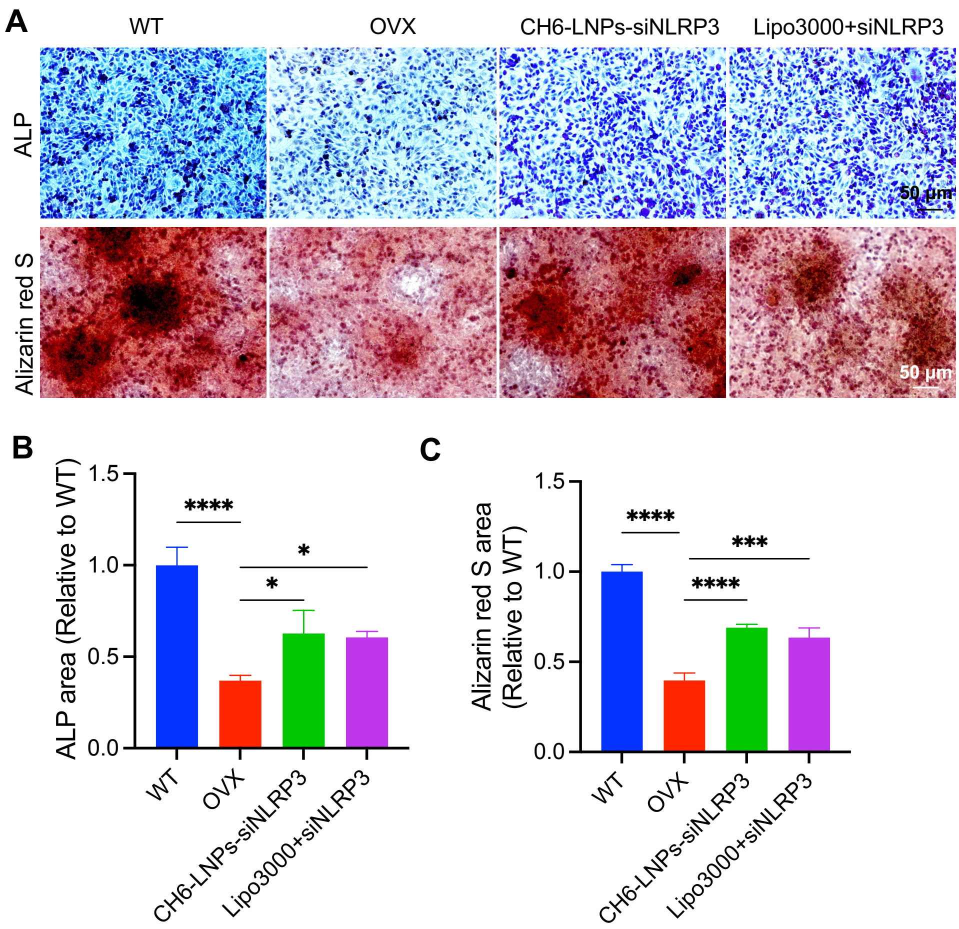

Figure S5. Alizarin red S and ALP staining analyzed the differentiation of osteoblasts. (A) Representative images of Alizarin red S and ALP staining in osteoblasts isolated from OVX treated with CH6-LNPs-siNLRP3 and siNLRP3 carried by Lipo3000. Scale bar represents 50 μm. **(B, C)** The ALP (B) and Alizarin red S (C) staining area of osteoblasts was quantified by Image J. Statistical significance was calculated by one-way ANOVA followed by Dunnett's test (Compared with OVX, *$P < 0.05$, ***$P < 0.001$, ****$P < 0.0001$)

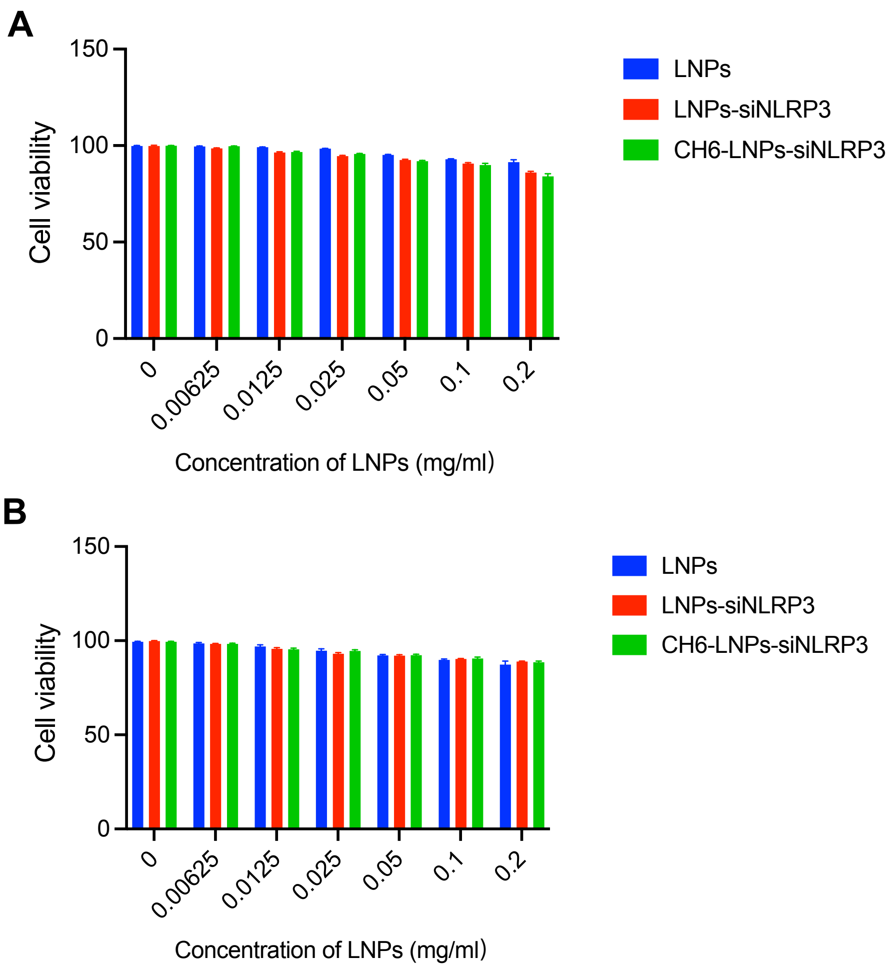

Figure S6. Toxicity of CH6-LNPs-siNLRP3 on osteoblasts and 293T cells. (A-B), Different concentrations of LNPs, LNPs-siNLRP3 and CH6-LNPs-siNLRP3 were added to the culture of osteoblasts (A) and 293T cells (B), respectively. Cell viability was measured by MTT assay after 72 h-incubation.

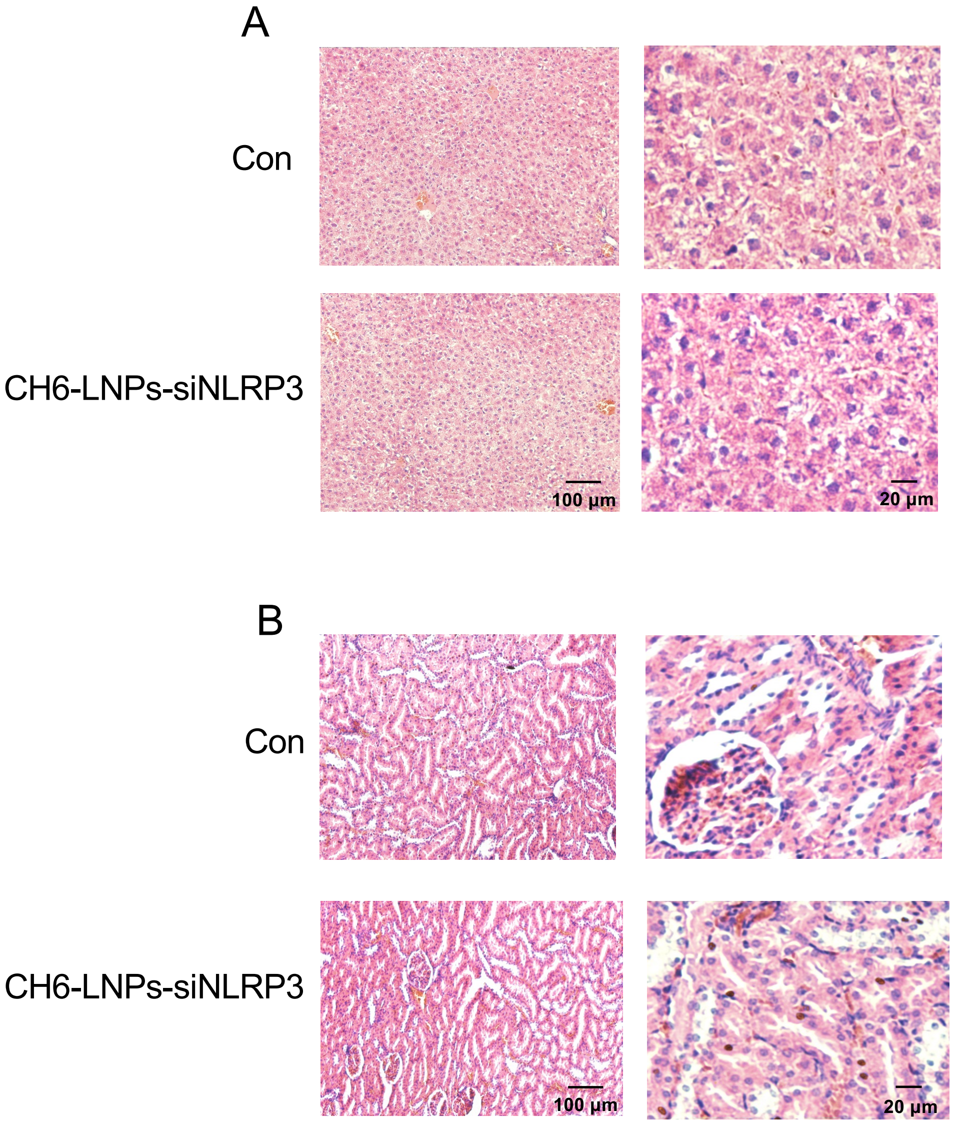

Figure S7. HE staining of liver and kidney. 6-week-old female SD rats were treated with CH6-LNPs-siNLRP3 via tail vein injections at a single dose of 1.0 mg/kg siNLRP3 encapsulated in LNPs-based carriers. Rats treated with PBS was used as a control. Livers **(A)** and kidneys **(B)** of rats were collected and analyzed by HE staining 7 days post injection.

A

CH6-LNPs-siNLRP3 (μg/ml)	Hemolysis rate (%)
2	0.90±0.09
10	4.30±0.08
50	3.50±0.08
100	4.06±0.09
200	1.78±0.13
400	3.59±0.05

B

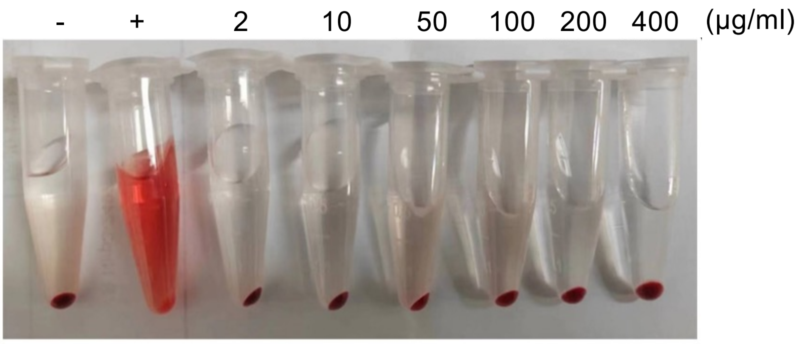

C

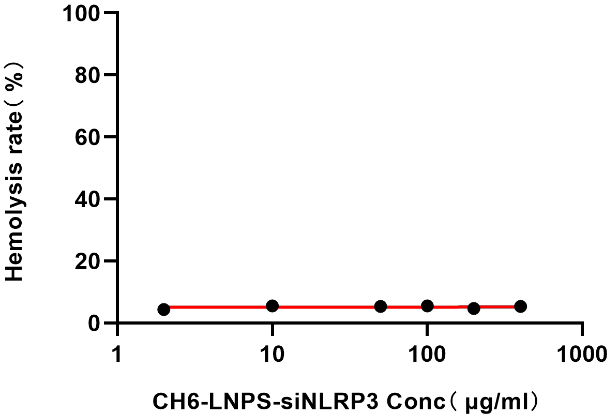

Figure S8. The biocompatibility of the CH6-LNPs-siNLRP3. (A) The biocompatibility of CH6-LNPs-siNLRP3 was analyzed by hemolysis assay. 2, 10, 50, 100, 200 or 400 μg/ml of CH6-LNPs-siNLRP3 was incubated with 2% red blood cell suspension at room temperature for 4 h, and then was centrifugated for 5 min at 10016g, 100 μl of the supernatant of all samples was transferred to a 96-well plate and the absorbance was measured. (B) Representative images of different concentrations of CH6-LNPs-siNLRP3 incubated with 2% red blood cell suspension. (C) Hemolysis rate cure of CH6-LNPs-siNLRP3.

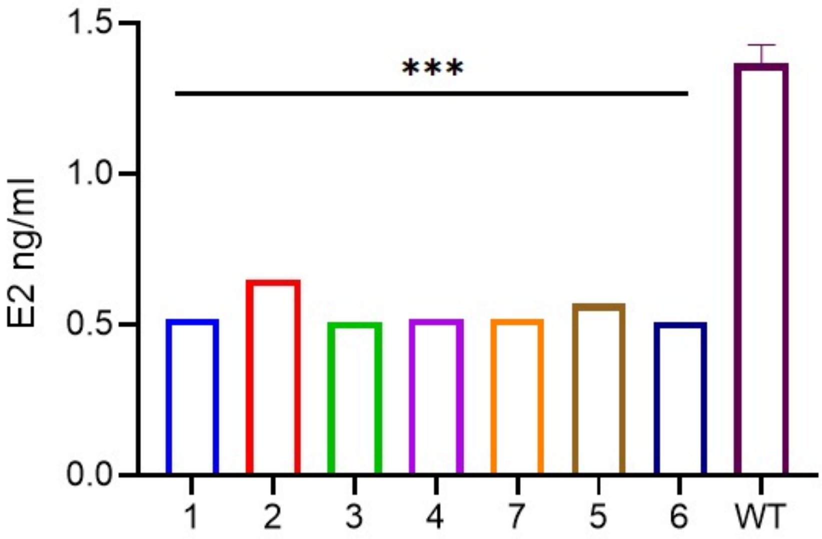

Figure S9. The E2 levels in OVX rats. After OVX operations, six rats were randomly selected and their E2 levels were analyzed by ELISA. Statistical significance was calculated by Student t-test (Compared with WT, ****P* < 0.001)

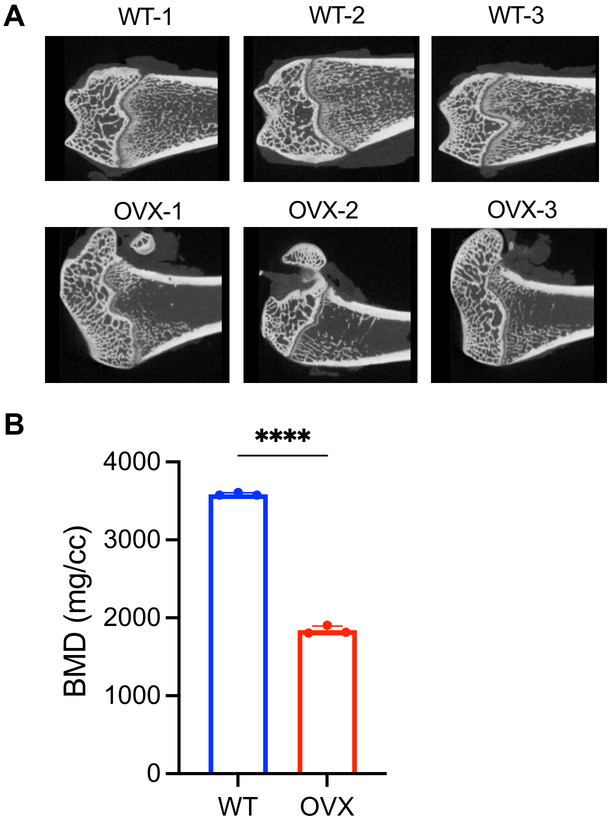

Figure S10. Micro-CT of OVX rats. (A) 8 weeks after bilateral OVX surgery, the femur of OVX rats was detected by micro-CT. **(B)** The BMD of OVX rats were analyzed. n = 3 per group. Statistical significance was calculated by Student t-test (Compared with WT, ****P < 0.0001)

Table S1. Demographic details of the plasma used in this study

Case	Age	Gender	Clinical diagnosis	Case	Age	Gender	Clinical diagnosis
1	62	F	PMOP	43	56	F	PMOP
2	70	F	PMOP	44	65	F	PMOP
3	66	F	PMOP	45	55	F	PMOP
4	67	F	PMOP	46	57	F	PMOP
5	56	F	PMOP	47	52	F	PMOP
6	66	F	PMOP	48	54	F	PMOP
7	68	F	PMOP	49	74	F	PMOP
8	73	F	PMOP	50	46	F	PMOP
9	52	F	PMOP	51	57	F	PMOP
10	52	F	PMOP	52	56	F	PMOP
11	62	F	PMOP	53	58	F	PMOP
12	66	F	PMOP	54	72	F	PMOP
13	68	F	PMOP	55	73	F	PMOP
14	63	F	PMOP	56	71	F	Normal
15	68	F	PMOP	57	56	F	Normal
16	73	F	PMOP	58	69	F	Normal
17	60	F	PMOP	59	76	F	Normal
18	66	F	PMOP	60	55	F	Normal
19	69	F	PMOP	61	64	F	Normal
20	56	F	PMOP	62	73	F	Normal
21	58	F	PMOP	63	68	F	Normal
22	84	F	PMOP	64	58	F	Normal
23	64	F	PMOP	65	55	F	Normal
24	56	F	PMOP	66	61	F	Normal

25	80	F	PMOP	67	77	F	Normal
26	47	F	PMOP	68	76	F	Normal
27	62	F	PMOP	69	79	F	Normal
28	73	F	PMOP	70	75	F	Normal
29	53	F	PMOP	71	80	F	Normal
30	54	F	PMOP	72	80	F	Normal
31	52	F	PMOP	73	81	F	Normal
32	79	F	PMOP	74	61	F	Normal
33	62	F	PMOP	75	56	F	Normal
34	55	F	PMOP	76	44	F	Normal
35	58	F	PMOP	77	57	F	Normal
36	65	F	PMOP	78	51	F	Normal
37	69	F	PMOP	79	53	F	Normal
38	63	F	PMOP	80	46	F	Normal
39	59	F	PMOP	81	53	F	Normal
40	63	F	PMOP	82	50	F	Normal
41	70	F	PMOP	83	53	F	Normal
42	59	F	PMOP	84	46	F	Normal
